# Biomarkers of Depression among Adolescent Girls: BDNF and Epigenetics

**DOI:** 10.3390/ijms25063281

**Published:** 2024-03-14

**Authors:** Weronika Zwolińska, Karolina Bilska, Kateryna Tarhonska, Edyta Reszka, Maria Skibińska, Natalia Pytlińska, Agnieszka Słopień, Monika Dmitrzak-Węglarz

**Affiliations:** 1 Department of Child and Adolescent Psychiatry, Karol Jonscher Clinical Hospital, Poznan University of Medical Sciences, Szpitalna 27/33 St., 60-572 Poznan, Poland; weronika.zwolinska@student.ump.edu.pl (W.Z.); nataliapytlinska@ump.edu.pl (N.P.); agaslopien@ump.edu.pl (A.S.); 2 Department of Psychiatric Genetics, Medical Biology Center, Poznan University of Medical Sciences, Rokietnicka St. 8, 60-806 Poznan, Poland; kbilska@ump.edu.pl (K.B.); mariaski@ump.edu.pl (M.S.); 3 Department of Translational Research, Nofer Institute of Occupational Medicine, 91-348 Łódź, Poland; kateryna.tarhonska@imp.lodz.pl (K.T.); edyta.reszka@imp.lodz.pl (E.R.)

**Keywords:** mood disorders, depression, adolescence, child psychiatry, biomarkers, brain-derived neurotrophic factor, epigenetics, DNA methylation

## Abstract

Alterations in brain-derived neurotrophic factor (BDNF) expression have been suggested to mediate the influence of environmental factors on the emergence of depression through epigenetic modifications. However, research on this subject in the developmental population is lacking and the pathophysiology of adolescent depression remains unclear. We aimed to investigate the alterations in *BDNF* expression and global DNA methylation in depression among adolescent girls. Thirty female inpatients with the initial diagnosis of depression were assessed before and after the period of antidepressant treatment and compared with thirty age-matched healthy controls. The assessment involved BDNF and proBDNF serum levels, the *BDNF* gene exon IV promoter methylation, and global DNA methylation. The methylation level in the *BDNF* gene exon IV promoter was significantly lower in the studied group compared with the control and correlated negatively with the severity of depression. The test distinguished the studied group from the controls with a sensitivity of 37% and specificity of 90%. The differences were no longer present after the period of antidepressant treatment. No differences in the global DNA methylation, BDNF, and proBDNF levels were found. We concluded that decreased methylation in the *BDNF* exon IV promoter could be considered as a biomarker of a depression state among adolescent girls.

## 1. Introduction

Major depressive disorder (MDD) occurs throughout one’s lifespan, with the most probable period for the onset of the first episode extending from adolescence to middle age [1]. It is mainly characterized by a depressed mood, anhedonia, and loss of energy. Other symptoms include sleep disturbances, appetite loss, difficulty concentrating, feelings of worthlessness, psychomotor retardation, and suicidal ideation [2]. Recent studies have reported that up to 34% of adolescents globally may be at risk of developing clinical depression [3]. Adolescents suffering from depression are prone to substance abuse, long-term psychosocial impairment in adulthood, and suicidal attempts [4]. The latter constitutes the fourth leading cause of death among 15–29-year-olds [5]. In practice, the detection and diagnosis of depression among adolescents often pose challenges for clinicians due to its various presentations, which differ from symptoms presented by adult patients [4,6,7]. Despite the significant prevalence, the pathophysiology of this condition remains vague, and the treatment outcomes are unsatisfying [8].

MDD is known to have heterogeneous etiology with the interaction between genetic and environmental factors underlying the manifestation of symptoms [1,9]. Among the various theories explaining the pathophysiology of depression, neurotrophic theory is one of the most commonly investigated. It assumes that environmental stress factors decrease the synthesis of brain-derived neurotrophic factor (BDNF) in the areas of the brain involved in cognitive functions and mood regulation, resulting in decreased synaptic plasticity, decreased synaptic transmission, and increased neuronal degeneration [10]. These impairments lead to specific structural changes in the brain such as atrophy of the prefrontal cortex and hippocampal shrinkage, which underlie the development of depressive symptoms [11,12]. Neurotrophic theory is supported by studies showing a decreased level of BDNF in the postmortem brain samples of patients suffering from MDD [13,14]. Considering these discoveries, studies have focused on the potential of using BDNF as a specific indicator of the depression state and recovery. Studies performed on the adult population have coherently demonstrated that the BDNF circulating level is significantly lower in the blood samples of depressed patients and that effective antidepressant treatment can reverse this effect, making BDNF a potential biomarker of depression and recovery [15,16].

BDNF is first synthesized as a precursor protein (proBDNF) that is further extracellularly cleaved into its mature form by proteases. Interestingly, both particles are biologically active and have been shown to elicit opposing effects by two principal transmembrane-receptor signaling systems: mature BDNF binds tropomyosin-related kinase B receptor (TrkB), which promotes long-term potentiation and stimulates neuronal survival, while proBDNF evokes long-term neuronal depression through the p75^NTR^ receptor, which results in neuronal death [17,18]. Therefore, proBDNF cleavage constitutes an important mechanism regulating the opposing effects of BDNF and proBDNF on neuronal survival. The failure in this regulation is now considered one of the possible mechanisms responsible for pathological hippocampal cell death associated with the pathogenesis of depression [17,19]. In line with this hypothesis, studies have revealed increased serum proBDNF levels and increased expression of the p75^NTR^ receptor among adults with depression [20,21]. Hence, not only may the downregulation of BDNF be typical for depression, but the upregulation of its precursor could also be considered a biomarker of the depression state.

Changes in BDNF expression in depression have also been identified on the molecular level in the epigenetic modifications of the *BDNF* gene [22]. Epigenetic modifications include chemical reactions such as methylation, acetylation, and phosphorylation, which modify the expression of genes. DNA methylation has been implicated in psychiatric disorders as a mechanism by which experiencing environmental stress alters neuronal gene transcription. *BDNF* gene expression is controlled by nine promoters, each regulating the expression of distinct BDNF transcripts that contribute to a region-specific BDNF effect in the brain [11,23]. These promoters are stimulated in a developmental, tissue specific, and activity-dependent matter [18]. Neuronal activity-dependent activation of the *BDNF* gene in the hippocampus—an area of the brain known to be involved in the pathophysiology of depression—is mediated by decreased methylation of *BDNF* promoter IV, among other mechanisms [24,25]. Therefore, alterations in the methylation of this promoter have been investigated as a potential biomarker in adult and late-life depression [22,26]. However, to date, there has been no study investigating the methylation of *BDNF* promoter IV among adolescent patients suffering from depression. It seems particularly interesting to investigate this issue in youth depression since exon IV is expressed differentially throughout development, with its transcripts increasing gradually during embryonic and postnatal development and decreasing slightly in the adult brain [24]. Apart from analyzing the methylation of specific gene promoters, studies on adults have revealed that DNA methylation in depression is globally altered [27,28]. It has been reported that alterations in global DNA methylation in adolescence are associated with early-life adversity, which is also a well-known risk factor of depression [29]. However, no study has investigated the correlation between global DNA methylation and depression in the youth population.

Although epigenetic alterations as well as changes in *BDNF* expression have been widely investigated as potential biomarkers in adult depression, there is a significant paucity of research on this subject in the youth population [30]. Studies on depression involving the pediatric population seem essential to comprehend the natural course of the disease and eliminate potentially confounding factors present in adulthood such as several recurrent episodes, comorbidities, and a history of medication. Since BDNF has been hypothesized to regulate fear circuit plasticity during a sensitive period of early adolescence, the alterations in BDNF expression have been suggested to have a persistent impact on fear-related disorders later in life [31]. Therefore, early changes in BDNF expression could be characteristic of depression susceptibility as well as a depression state in adolescence. Nevertheless, current knowledge on the relationship between BDNF and depression in adolescents is still inconsistent, with contrasting results among different studies [32,33]. To date, no study has included the combined analysis of BDNF and proBDNF serum levels as well as *BDNF* epigenetic modifications in the population of adolescent patients with depressive symptoms. We hypothesize that adolescents suffering from depressive symptoms might present with altered *BDNF* expression when compared with their healthy peers. Following the results of adult studies, we assumed a decreased BDNF serum level, increased proBDNF serum level, and increased *BDNF* exon IV promoter methylation profile to characterize adolescent depression.

This study aimed to investigate the global DNA methylation and *BDNF* expression including the *BDNF* exon IV promoter methylation level, proBDNF, and BDNF serum levels in the group of adolescent patients treated for the first episode of depression in order to assess its usefulness in the diagnostics of adolescent MDD.

## 2. Results

### 2.1. Recruitment

Forty-nine patients were identified to meet the criteria of inclusion in the study. Fifteen patients were excluded due to lack of consent to participate in the study (eight patients), lack of compliance in taking the medication (one patient), and lack of follow-up assessment after treatment (six patients). Finally, thirty-four patients were included in the study: thirty girls and four boys. Due to the significant disproportion between the sexes, only female patients were finally accepted in the analysis as a studied group. Thirty age-matched healthy girls were recruited as a control group. All participants were of Caucasian origin.

### 2.2. Clinical Characteristics

The characteristics of the studied group and control is presented in Table 1. We identified no significant differences between the groups in terms of age (*p* = 0.373) and BMI (*p* = 0.371). The results of CDI-2 at t0 were significantly higher in the studied group when compared with healthy subjects (*p* < 0.000001). The treatment period until the second assessment at t1 took 7.27 weeks on average. Out of 30 patients, 13 were classified as ‘responders’ and 17 as ‘non-responders’ during the assessment at t1. As expected, the responders had significantly lower CDI-2 and HDRS results when compared with the non-responders (*p* = 0.00004 and *p* = 00002). Responders at t1 did not significantly differ from the healthy control regarding the CDI-2 results (*p* = 0.514), while the non-responders still had significantly higher CDI-2 results (*p* = 0.000003).

### 2.3. Biochemical and Molecular Results

The results of the mean serum levels of BDNF and proBDNF as well as the *BDNF* promoter methylation index (MI) and global methylation within the studied group at t0 and t1 and the healthy controls are presented in Table 1. During the molecular analysis of the control group, we failed to achieve the results in one participant for *BDNF* MI (*n* = 29), one participant for BDNF serum level (*n* = 29), three participants for global methylation level (*n* = 27), and six participants for the proBDNF serum analysis (*n* = 24). The proBDNF serum result was also missing in the studied group in two participants at t0 and five participants at t1. The results of the tests comparing the differences between the groups are summarized in Table 2 including the post hoc analysis of the achieved test power.

#### 2.3.1. BDNF and proBDNF Serum Levels

**Figure 1:**
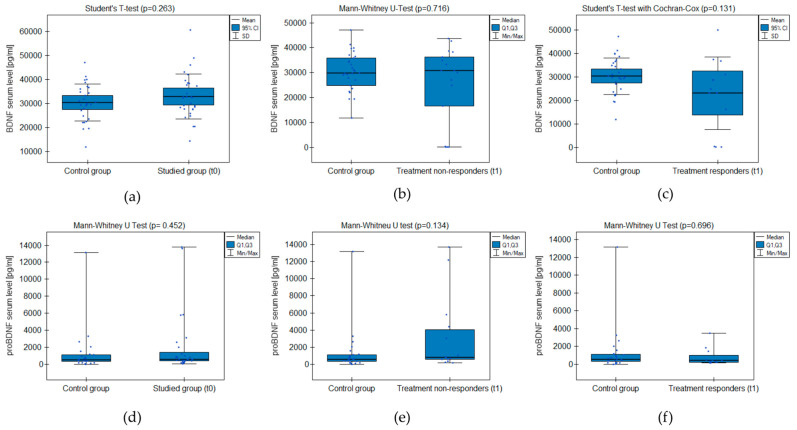
BDNF and proBDNF serum level comparison between the groups: (**a**,**d**) studied group before the treatment (t0) and healthy control; (**b**,**e**) treatment non-responders after the minimum 6 weeks of treatment (t1) and healthy control; (**c**,**f**) treatment responders after the minimum 6 weeks of treatment (t1) and healthy control.

The analysis did not prove significant differences between the groups’ BDNF and proBDNF serum levels (Figure 1). The test power was accurate in excluding the probability of II-type error when comparing serum BDNF levels between the studied group and control but not proBDNF (Table 2). There was no significant correlation between the level of depressive symptoms measured with CDI-2 and the BDNF/proBDNF serum levels within the whole sample.

#### 2.3.2. BDNF Gene Promoter Methylation

**Figure 2:**
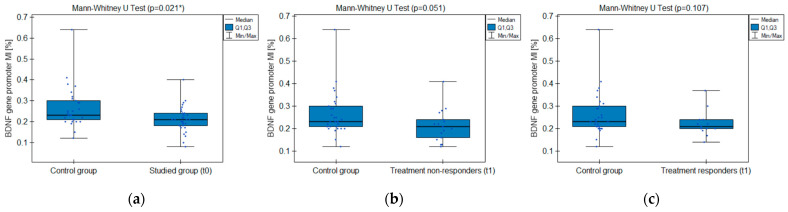
*BDNF* gene exon IV promoter methylation index (MI) comparison between the groups: (**a**) studied group before the treatment (t0) and healthy control; (**b**) treatment non-responders after the minimum 6 weeks of treatment (t1) and healthy control; (**c**) treatment responders after the minimum 6 weeks of treatment (t1) and healthy control. * Statistically significant difference (*p* < 0.05).

**Figure 3:**
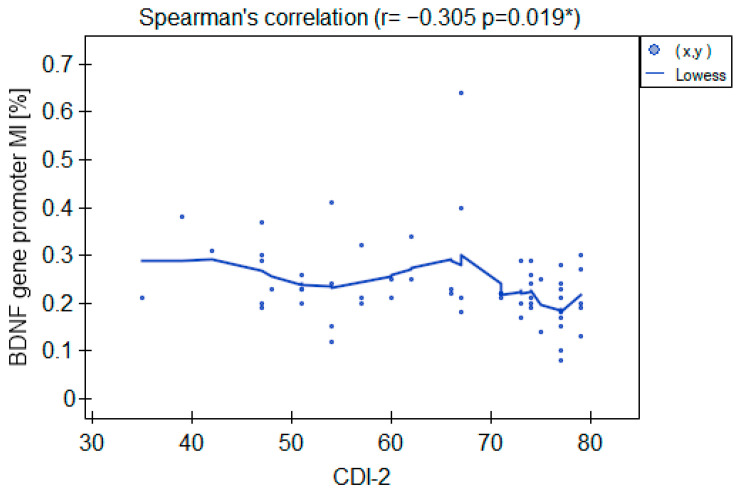
Correlation between the *BDNF* gene exon IV promoter’s MI and CDI-2 results in the whole sample. * Statistically significant (*p* < 0.05).

**Figure 4:**
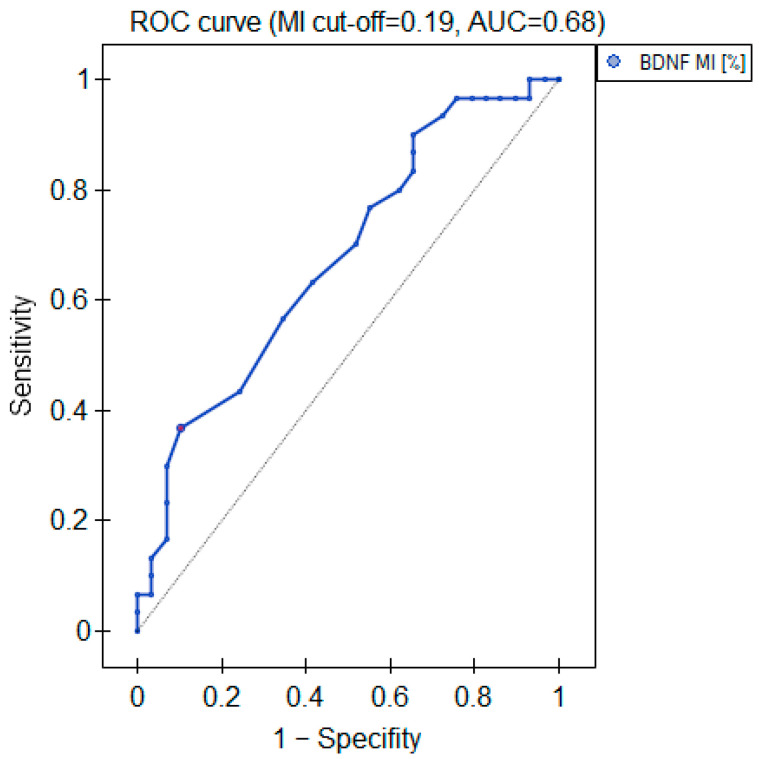
Diagnostic parameters of the *BDNF* gene exon IV promoter’s MI.

The MI within the promoter of the *BDNF* gene (exon IV) for the first primer version was found to be significantly lower within the group of patients with MDD symptoms before the treatment when compared with the healthy subjects (Figure 2a). There was a significant negative correlation between MI and the level of depressive symptoms within the whole sample (Figure 3). The association between MI and the presence of depression was also significant when controlled for age and BMI in the logistic regression model (β = −9.6; *p* = 0.031). The diagnostic ability of the test is presented as a ROC curve in Figure 4. The analysis revealed that at the threshold of MI = 0.19, the test distinguished between the subjects suffering from MDD symptoms and healthy controls with a sensitivity of 37% and specificity of 90% (PPV = 79%; NPV = 58%). After treatment, the responders’ MI did not significantly differ from the control (Figure 2c). The MI among non-responders was still lower than that in the controls (Figure 2b) at the threshold of statistical significance (*p* = 0.051). There were no significant differences between the groups when the second primer version was used in the analysis. There were no significant correlations between the methylation levels in the *BDNF* exon IV and proBDNF/BDNF serum levels for both primers.

#### 2.3.3. Global DNA Methylation (%-5mc)

The analysis did not prove any significant differences in the global DNA methylation between the groups (Table 2). There was no significant correlation between the level of depressive symptoms measured with CDI-2 and %-5mc within the whole sample.

## 3. Discussion

We found that adolescent girls suffering from depressive symptoms exhibited lower methylation levels in the *BDNF* gene exon IV promoter region when compared with their healthy peers. Consistently, the methylation level correlated negatively with the severity of depressive symptoms across the whole group. The differences in *BDNF* promoter MI were no longer present after the antidepressant treatment; however, compared to the controls, non-responders still presented a lower methylation index, falling short of statistical significance. We found no differences in the global DNA methylation between healthy and depressed subjects.

To the authors’ knowledge, ours is the first study to investigate the methylation of the *BDNF* exon IV promoter among human depressed adolescents. Consistent with our findings, studies on human adolescents investigating epigenetic changes in the BDNF gene following the experience of environmental stress, which is a well-known factor conducive to depression, reported decreased DNA methylation in the *BDNF* gene IV promoter [34]. Interestingly, the mother’s prenatal depressive symptoms were proven to predict decreased *BDNF* IV DNA methylation in their infants [35]. Hence, it seems plausible that decreased methylation of the BDNF exon IV promoter constitutes a biomarker of adolescent depression state as well as susceptibility. In such cases, it might be considered a useful tool for the detection of groups prone to disease development in order to introduce early preventive measures. In our study, the adolescents resistant to antidepressant treatment still presented lower MI when compared with the healthy subjects, contrary to the group of responders. This notion further supports the hypothesis that decreased *BDNF* IV DNA methylation can be associated with depressive symptoms. However, our study revealed the limited utility of MI in the exon IV promoter of the *BDNF* gene as a single biomarker of adolescent depression due to the low sensitivity (37%). On the other hand, the high specificity of the test (90%) might be promising when combined with other, more sensitive, diagnostics.

So far, clinical studies on *BDNF* promoter methylation in depression have only been performed on populations of adult patients [22]. Contrary to our findings, most of these studies reported no differences in the methylation status in the *BDNF* exon IV promoter between depressed patients and the healthy controls [36,37]. Epigenetic alterations associated with adult depression were identified in exon I rather than in exon IV [36,37,38]. One of the hypotheses to explain these discrepancies could be that different methylation profiles of the *BDNF* gene are typical for depression in adolescence and adulthood since distinct promoters of the *BDNF* gene control the expression of BDNF in various parts of the brain according to the developmental stage [11,23,39,40]. Preclinical studies identified the promoter of exon IV as part of the *BDNF* gene that regulates the expression of BDNF dynamically throughout development. Specifically, de novo methylation of the *BDNF* promoter at exon IV represses BDNF transcript levels prenatally in the pallium and subpallium. As development proceeds, exon IV becomes demethylated in tissues with high levels of BDNF expression such as the hippocampus and cortex [39]. Hence, exon IV of the *BDNF* gene seems to play a crucial role in proper brain development and therefore, alterations in the methylation of its promoter might be more associated with psychopathology in adolescence than in adulthood.

Counterintuitively, we found hypomethylation in the *BDNF* gene to be associated with depression rather than hypermethylation, which is known to repress protein expression. One plausible explanation could be that the observed hypomethylation in the *BDNF* exon IV promoter is rather a compensatory mechanism occurring due to another pathological process directly influencing the emergence of depressive symptoms. It could be possible that the pathogenesis of depression in adolescence is related to a different mechanism than that described in neurotrophic theory, and the observed hypomethylation of the *BDNF* exon IV promoter is a compensation that might no longer be efficient in adult age, when *BDNF* expression physiologically decreases [24,31,41]. In such case, it might be interesting to investigate whether unchanged methylation in the *BDNF* exon IV promoter in adolescent depression could be associated with worse disease course in the future and therefore verify the usefulness of decreased methylation in the *BDNF* exon IV promoter as a prognostic biomarker of depression. On the other hand, hypomethylation might result in the enhanced expression of the BDNF precursor, proBDNF, which is known to be conducive to neurodegeneration and depression [18,20,21]. In our study, we found no differences in the proBDNF serum levels between the studied group and the control. However, we have to acknowledge the fact that our analysis of the proBDNF serum level might have been too underpowered to prove any differences between the groups. More studies on larger groups are needed to investigate the role of proBDNF in the pathogenesis of adolescent depression and verify the possibility of using proBDNF as a biomarker of depression in the developmental age.

To our knowledge, ours is the first study to test both proBDNF and BDNF serum levels among adolescents with depression. It is particularly important since some discrepancies in previous studies might have resulted from not distinguishing between the mature BDNF and proBDNF in the analysis [42,43]. Regarding the BDNF serum levels, our results with adequate power indicate no significant correlations between the BDNF peripheral level and depressive symptoms in adolescent girls, which is in line with some of the previous reports on this subject [44,45,46]. However, our results remain in contrast with most studies on BDNF serum levels in the adult population, in which a decrease in serum BDNF has been widely associated with the presence of depressive symptoms [15,33,47]. It could be possible that a decrease in the BDNF serum level characterizes depression later in life, while it is not so evident in the developmental age. Physiologically, endogenous levels of BDNF rise dramatically in early adolescence and then gradually decrease with age [31,41]. Preclinical studies have revealed that animals subjected to early stress examined at a younger age exhibited enhanced BDNF levels as opposed to middle-aged animals [40]. Taking into consideration the different patterns of BDNF expression in response to stress during human development, longitudinal studies are needed to verify whether there are any differences in the dynamics of BDNF serum changes across adolescence in stress-related disorders. Remarkably, the peripheral BDNF level among depressed adolescents has been suggested to be gender-specific, which should be taken into consideration when interpreting our results. Several studies have reported identifying lower BDNF serum concentrations solely among depressed adolescent boys, but not among depressed girls [32,48,49]. Indeed, studies on rodent models report that BDNF expression is significantly increased in response to estrogen, which might potentially explain the lack of differences in the BDNF serum levels between healthy and depressed girls [50,51]. Therefore, more gender-specific studies are needed to verify whether the BDNF serum level might be applicable as a biomarker of depression in adolescence.

Some limitations should be considered when interpreting the results of our study. Firstly, the studied group was too small to reach adequate statistical power for some tests (specifically proBDNF and the global DNA methylation level). We should also acknowledge a possible bias in the participant selection and limited analytical sensitivity, in other words, the smallest amount of the tested substance that can be reliably detected using the applied tests (245 pg/mL for BDNF and 4.69 pg/mL for proBDNF). Secondly, we were not able to control every single known determinant of the serum BDNF level, which might have potentially influenced the results (for instance, seasonality or menstrual cycle status) [52]. Similarly, the methylation level could have been influenced by some internal (such as hormones, genetic allelic variations, blood composition) or external factors (such as diet, physical activity, stress or toxins exposure) [53,54,55]. Although our study only focused on female adolescents, this limitation may be considered an advantage, given the different course of the disease between genders [56].

## 4. Materials and Methods

### 4.1. Ethical Declaration

The study followed the ethical standards established in the Declaration of Helsinki and was reviewed and approved by the Bioethics Committee of the Poznan University of Medical Sciences. All participants’ legal guardians and patients above 13 years of age gave their written informed consent to participate in the study.

### 4.2. Participants

The studied group was recruited between January 2021 and April 2023 among the inpatients of the Child and Adolescent Psychiatry Clinic in Poznan, Poland. Throughout this period, all patients admitted to the inpatient psychiatric unit of the Child and Adolescent Psychiatry Clinic in Poznan were actively screened for the inclusion criteria, which involved: age 11–17, admitted with the initial diagnosis of the first episode of depression with no history of psychiatric treatment, no acute somatic disease (such as infection), no chronic somatic disease, no chronic medical treatment, no history of addiction, and no organic causes of depressive symptoms.

For inclusion in the study, at least a moderate level of depressive symptoms according to ICD-10 criteria for depression must have been present, meaning at least two of the key symptoms (depressed mood, loss of interest in everyday activities, reduction in energy), plus at least three of the remaining seven symptoms (disturbed sleep, poor concentration/indecisiveness, low self-confidence, poor/increased appetite, suicidal thoughts or acts, agitation/slowing of movements, guilt or self-blame) persisting over a period of two weeks [57]. Additionally, clinical indications to introduce the treatment with selective serotonin reuptake inhibitor (SSRI) must have been present in line with the NICE guidelines [58]. The decision regarding the initial diagnosis and evaluation of the indications for the antidepressant treatment were made by the patient’s leading child psychiatrist based on the examination of the patient, one-week observation of the patient’s behavior on the ward, and detailed interview with a parent. The participation in the study did not influence any treatment decisions.

**Figure 5:**
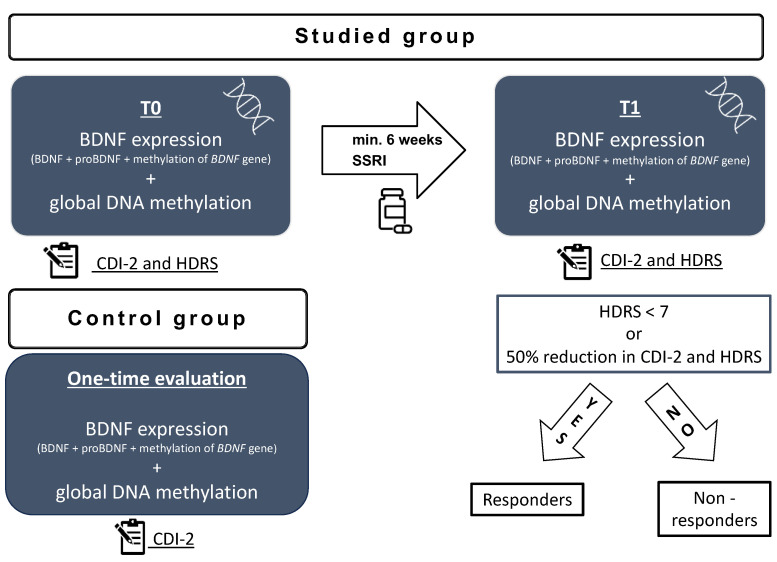
Study scheme: t0—on admission to the hospital before treatment; t1—after a minimum 6 weeks of antidepressant treatment; HDRS—Hamilton Depression Rating Scale; CDI-2—Children’s Depression Inventory; BDNF—brain derived neurotrophic factor; SSRI—selective serotonin reuptake inhibitor.

Clinical and biochemical assessment was made at two time points for the studied group (Figure 5): after admission to the psychiatric ward but before introducing the antidepressant treatment (t0) and after the minimum of 6 weeks of antidepressant treatment (t1). According to clinical condition, if the patient improved with treatment, they were discharged home and scheduled for a second assessment after the minimum 6 weeks of treatment. If the patient did not improve, the second assessment was performed after a minimum of 6 weeks of treatment on the psychiatric inpatient unit and before switching the treatment. The exclusion criteria involved mental retardation, substance abuse, the parent or patient’s consent to withdraw, failure to continue treatment with the SSRI throughout the minimum of 6 weeks, and failure to collect blood samples at any of the time points.

The control group was recruited by means of an announcement in the outpatient clinic. The inclusion criteria involved no depressive symptoms, no history of psychiatric disorders, no chronic somatic diseases, and no chronic pharmacological treatment. The participants were offered free basic blood test results for taking part in the study. The recruitment of the control group took place between January 2023 and April 2023. The volunteers were selected to match the age and sex of the studied group. The volunteers completed the CDI-2 self-report short form and were additionally interviewed by a child psychiatrist to screen for the presence of depressive symptoms. A detailed interview with a parent was conducted to exclude the presence of any somatic comorbidities. All participants’ legal guardians and children above 13 years of age gave their written informed consent to participate in the study. Clinical and biochemical assessment was made at one time point for the control group (Figure 5). The exclusion criteria involved any active disease, current infection, mental retardation, history of substance abuse, the parent or patient’s consent to withdraw, and a failure to collect blood samples.

A total sample size was calculated assuming error probability at the levels of α = 0.05 and 1 − β = 0.8. We calculated the minimum required sample size based on previous reports on the subject of BDNF serum levels among adolescents with depression [32] and proBDNF serum levels among adolescents with PTSD [59], since no study has previously investigated proBDNF among adolescents with depression. The analysis resulted in the minimum of twenty-six participants for BDNF and twenty-one for proBDNF to reach the above-mentioned statistical power.

### 4.3. Clinical Assessment

The severity of depressive symptoms was assessed at t0 and t1 by means of the 17-item Hamilton Depression Rating Scale (HDRS) and Children’s Depression Inventory 2 (CDI-2) short forms [60,61]. The HDRS is widely used in depression research for patient selection and follow-up [62]. CDI-2 is a self-report instrument standardized for assessing depressive symptoms in children and adolescents used by both practitioners and researchers [63]. It comprises 12 questions with three possible answers referring to the presence of depressive symptoms characteristic for the child population; for instance: ‘I make up my mind about things easily/it is hard to make up my mind about things/I cannot make up my mind about things’. The final score of the CDI-2 self-report is standardized for the children’s age and sex. The CDI-2 short form is a subjective tool for depression assessment, in contrast to HDRS, which is completed by the clinician based on visible symptoms. The clinical assessment of each participant was performed by the same trained child psychiatrist from the present authors’ group at both time points. Based on the results at t1, the patients were assigned to the group of ‘responders’ and ‘non-responders’. To be classified as a ‘responder’, a participant must have been assessed with <7 points in HDRS or at least a 50% reduction in symptoms in both HDRS and CDI-2 [64].

### 4.4. Biochemical Assessment

All participants had their blood samples taken to evaluate the *BDNF* expression (methylation in the *BDNF* promoter region, serum BDNF, and proBDNF) as well as global DNA methylation level (%5-mc). Two 5 mL peripheral blood samples of each fasting participant were collected into anticoagulant-free and EDTA tubes between 7 a.m. and 10 a.m.

#### 4.4.1. Global DNA Methylation

The DNA was extracted from 5 mL of EDTA anticoagulated whole blood using the salting-out method [65]. The 5-mC level in 50 ng of genomic DNA was quantified with a Colorimetric Methyl Flash Global DNA Methylation (5-mC) ELISA Easy Kit according to the manufacturer’s instructions (Epigentec, Farmingdale, NY, USA). The absorbance was measured on a Multiskan GO (Thermo Fisher Scientific, Waltham, MA, USA).

#### 4.4.2. BDNF Methylation Analysis

The level of methylation in the *BDNF* (exon IV) promoter region was analyzed using quantitative methylation-specific real-time PCR (qMS-PCR). DNA sequence regions that obtained the promoter region of exon IV (27,722,850–27,723,477 Homo sapiens chromosome 11, GRCh37.p13) were used to design two versions of the primers (Table 3). The primers were designed with Methyl Primer Express™ Software v1.0 (Applied Biosystems, Waltham, MA, USA). For a detailed description of the primers, see Table S1 in the Supplementary Materials. Isolated genomic DNA and CpGenome Human Methylated & Non-Methylated DNA Standard Set (Sigma-Aldrich, Merck KGaA, Darmstadt, Germany) was converted using a sodium bisulfite kit. Chemical modification of 500 ng of genomic DNA and standards was performed using an EZ DNA Methylation Gold Kit™ (Zymo Research, Irvine, CA, USA). After sodium bisulfite conversion, the percentage of methylation index (MI) was assessed by qPCR with two pairs of primers for the methylated and unmethylated promoter region of the *BDNF* gene with FastStart Essential DNA SYBR Green Master (Roche, Basel, Switzerland). The MI, expressed as a percentage of gene methylation (MI—%), was calculated for each sample using the following formula: MI = [1/(1 + 2^−(CtU-CtM)^)] × 100%, where CtM and CtU are derived from qMSP with primers for the methylated and unmethylated gene sequences, respectively.

#### 4.4.3. BDNF and proBDNF Serum Levels

After one hour of incubation, serum was separated by centrifugation, aliquoted, and stored at −70 °C until further analysis. Enzyme-linked immunosorbent assays were performed using BDNF_DuoSet Human/Mouse (cat. no DY248), proBDNF_DuoSet Human (cat. no DY3175), and ELISA Development Kit (R&D System, Minneapolis, MN, USA) according to the manufacturer’s instructions. Plates were blocked for three hours in reagent diluent (1% bovine serum albumin (BSA)/phosphate buffered saline (PBS)) and incubated overnight with 100 μL of the samples at 4 °C with shaking. Samples were diluted 1:120 for BDNF and 1:2 for proBDNF to fit the standard curve range. All plates were run within one week on the same kit lot by the same experienced operator. Standard curves for all analytes ranged from 1000 to 15.6 pg/mL for BDNF and from 4000 to 62.5 pg/mL for proBDNF. Intra-assay and inter-assay variability were <10% coefficient of variation (CV) and <15% CV, respectively.

### 4.5. Statistical Analysis

Statistical calculations were carried out using PQStat Software version 1.8.2.238. The distribution of the variables was studied by the Shapiro–Wilk test. A comparison of two unpaired groups was performed using the Student’s *T*-test (for the data that followed normal distribution) or Mann–Whitney U test (for non-parametric variables). The Spearman’s rank correlation coefficient was applied to assess the relationship between the clinical symptoms and analyzed variables. A multivariate logistic regression analysis was performed to verify the association between the analyzed factors and the presence of depression including the potential confounding factors (age, BMI). We performed ROC analysis to calculate the area under the curve (AUC) and determine the most appropriate cut-off for the predictive value of potential biomarkers. The minimum required sample size and post hoc statistical power analyses were performed using G*Power 3.1 Software. The significance level was set at α < 0.05 for all analyses.

## 5. Conclusions

Decreased methylation in the *BDNF* exon IV promoter should be considered as one of the possible biomarkers of a depression state among adolescent girls. More studies are needed to verify this hypothesis and comprehend the role of altered *BDNF* expression in developmental age depression. Future studies should involve a longitudinal analysis of *BDNF* expression in depression across adolescence and adulthood in order to understand the pathogenesis and natural course of the disease.

## Figures and Tables

**Table 1:** Characteristics of the studied groups.

	Studied Group (t0)	Responders (t1)	Non-Responders (t1)	Controls
n	30	13	17	30
Age [years]	13.07 (±1.26)	12.85 (±0.99)	13.24 (±1.44)	13.43 (±1.85)
Sex	females	females	females	females
BMI [kg/m^2^]	20.89 (±3.47)	21.51 (±2.44)	20.41 (±4.10)	20.46 (±4.38)
HDRS	20 (13–30)	4 (1–9)	12 (8–24)	−
CDI-2	74.5 (60–79)	54 (47–68)	74 (60–79)	54 (35–79)
Time of treatment [weeks]	7.27 (±1.41)	7.46 (±1.39)	7.12 (±1.45)	−
BDNF [pg/mL]	32,890 (±9352)	23,114 (±15,416)	25,247 (±15,712)	30,355 (±7763)
proBDNF [pg/mL]	2068 (±3608)	856 (±1027)	3180 (±4470)	1338 (±2649)
*BDNF* promoter MI [%] *	0.212 (±0.063)	0.223 (±0.058)	0.214 (±0.073)	0.264 (±0.099)
Global DNA methylation [%]	0.597 (±0.290)	0.479 (±0.365)	0.613 (±0.464)	0.520 (±0.231)

Continuous variables are presented as the mean and standard deviation; ordinal variables are presented as the median and range; t0—on admission to the hospital before treatment; t1—after minimum 6 weeks of antidepressant treatment; BMI—body mass index; HDRS—Hamilton Depression Rating Scale; CDI-2—Children’s Depression Inventory; BDNF—brain derived neurotrophic factor; MI—methylation index; * using primer version no. 1.

**Table 2:** Summary of the BDNF, proBDNF, *BDNF* promoter MI, and global DNA methylation comparison between the groups.

		Studied Group (t0) vs. Control	Responders (t1) vs. Control	Non-Responders (t1) vs. Control
BDNF	*p*-value	0.263	0.131	0.716
	test power	95%	95%	39%
proBDNF	*p*-value	0.452	0.696	0.134
	test power	20%	17%	43%
*BDNF* promoter MI (exon IV)	*p*-value	0.021 *	0.107	0.051
	test power	77%	44%	57%
Global DNA methylation	*p*-value	0.277	0.669	0.515
	test power	29%	10%	20%

* Statistically significant difference (*p* < 0.05). t0—on admission to the hospital before treatment; t1—after minimum 6 weeks of antidepressant treatment; BDNF—brain derived neurotrophic factor; MI—methylation index.

**Table 3:** Description of the primers.

Primer Version	Forward Methylated DNA (MF)	Reverse Methylated DNA (MR)	Forward Unmethylated DNA (UF)	Reverse Unmethylated DNA (UR)
1	AGCGAGAGTAGTTTTTTTCGC	CATATAACAACGCACGTCAAA	GGTAGTGAGAGTAGTTTTTTTTGT	TCATATAACAACACACATCAAAAC
2	TGTATGGCGGAGGTAATATTC	AAACTACTCTCGCTACCGCT	ATTGTATGGTGGAGGTAATATTT	AAAAACTACTCTCACTACCACT

## Data Availability

Dataset available on request from the authors.

## Supplementary Materials

The following supporting information can be downloaded at: https://www.mdpi.com/article/10.3390/ijms25063281/s1.
