# Supplementary material for: Biomarkers of Depression among Adolescent Girls: BDNF and Epigenetics
Source: Int J Mol Sci. 2024 Mar 14;25(6):3281. doi: 10.3390/ijms25063281 (PMC10970207; doi:10.3390/ijms25063281)
Supplement: Supplementary file 1 [file ijms-25-03281-s001.zip › ijms-2880785-supplementary.pdf]

Table S1. Description of the primers

<table>
<tr><th>Primer version 1</th><th>Primer version 2</th></tr>
<tr><td colspan="2">Forward Primer for Methylated DNA (MF)</td></tr>
<tr><td>5′ AGCGAGAGTAGTTTTTTTCGC 3′</td><td>5′ TGTATGGCGGAGGTAATATTC 3′</td></tr>
<tr><td colspan="2">Reverse Primer for Methylated DNA (MR)</td></tr>
<tr><td>5′ CATATAACAACGCACGTCAAA 3′</td><td>5′ AAACTACTCTCGCTACCGCT 3′</td></tr>
<tr><td colspan="2">Forward Primer for Unmethylated DNA (UF)</td></tr>
<tr><td>5′ GGTAGTGAGAGTAGTTTTTTTGT 3′</td><td>5′ ATTGTATGGTGGAGGTAATATTT 3′</td></tr>
<tr><td colspan="2">Reverse Primer for Unmethylated DNA (UR)</td></tr>
<tr><td>5′ TCATATAACAACACACATCAAAAC 3′</td><td>5′ AAAAACTACTCTCACTACCACT 3′</td></tr>
<tr><td colspan="2">Promoter sequence region</td></tr>
<tr><td>>ref|NC_000011.9|:27723083-27723477 Homo sapiens chromosome 11, GRCh37.p13 Primary Assembly</td><td>>ref|NC_000011.9|:27722850-27723394 Homo sapiens chromosome 11, GRCh37.p13 Primary Assembly</td></tr>
<tr><td colspan="2">gDNA Sequence</td></tr>
<tr><td>5′
TCCATTTGATCTCGGCAGAGGCAGGGAGATTTC
ATGCTAGTTCGCCGGGGGGAGCGGCAGCGAGA
GCAGCCCTCTCCGCGGTGAATGGGAAAGTGGGT
GGGAGTCCACGAGAGGGCTCCACGGTGCCTTG
ACGTGCGCTGTCATATGATACCTCCGCTGCCTC
GAAATAGACACTCTAGTGCACGAATTACCAGA
ATCAAAATTCAGCGCATTTAAAATGATACATCT
TTTATTAGAAGAGTTCCGTTCCAGGGCATTGCAT
GCTTTTGCAGATGTTTTCACTTCCAGCCCCAGCA
AACACACGTATAAGCTAACCCTTTTAATAACGA
ACCAGGGCAGCCAAGATAAATAAAAAGTCTTC
TGCTTTAACCAGAGTGGGGGTAGGTGATTCGAG
G 3′</td><td>5′
AGTCACTACTTGTCAAAGTAACCATCAAGGCAGC
TGCTCCGGGAAAGACTTCGGCCCCAAAACTCCCA
CACTCTATTATTTTTTCACGTTCCCTTCGCTTAATT
AAAGGGGGGAGGGGGCGCGAGTCTTTGGTGCCC
GGTATGTACTCCTTCTGTTCTGCAGCAAAGAAGTT
AAATTATTGATAGTGGAAATTGCATGGCGGAGGT
AATACTCGCACCCCATCAGCGAGAAGCTCCATTT
GATCTCGGCAGAGGCAGGGAGATTTCATGCTAGT
TCGCCGGGGGGAGCGGCAGCGAGAGCAGCCCTC
TCCGCGGTGAATGGGAAAGTGGGTGGGAGTCCA
CGAGAGGGCTCCACGGTGCCTTGACGTGCGCTGT
CATATGATACCTCCGCTGCCTCGAAATAGACACT
CTAGTGCACGAATTACCAGAATCAAAATTCAGCG
CATTTAAAATGATACATCTTTTATTAGAAGAGTTC
CGTTCCAGGGCATTGCATGCTTTTGCAGATGTTTT
CACTTCCAGCCCCAGCAAACACACGTATAAGCT
3′</td></tr>
<tr><td colspan="2">Bisulfite modification of DNA</td></tr>
<tr><td>5′ TTTATTTGATTTCGGTAGAGGTAGGGAGATTTT
ATGTTAGTTCGTCGGGGGGAGCGGTAGCGAGAG
TAGTTTTTTTCGCGGTGAATGGGAAAGTGGGTG
GGAGTTTACGAGAGGGTTTTACGGTGTTTTGAC
GTGCGTTGTTATATGATATTTTCGTTGTTTCGAA
ATAGATATTTTAGTGTACGAATTATTAGAATTAA
AATTTAGCGTATTTAAAATGATATATTTTTTATT
AGAAGAGTTTCGTTTTAGGGTATTGTATGTTTTT
GTAGATGTTTTTATTTTTAGTTTTAGTAAATATAC
GTATAAGTTAATTTTTTTAATAACGAATTAGGGT
AGTTAAGATAAATAAAAAGTTTTTTGTTTTAATT
AGAGTGGGGGTAGGTGATTCGAGG 3′</td><td>5′
AGTTATTATTTGTTAAAGTAATTATTAAGGTAGTT
GTTTCGGGAAAGATTTCGGTTTTAAAATTTTTATA
TTTTATTATTTTTTTACGTTTTTTTCGTTTAATTAAA
GGGGGGAGGGGGCGCGAGTTTTTGGTGTTCGGTA
TGTATTTTTTTTGTTTTGTAGTAAAGAAGTTAAATT
ATTGATAGTGGAAATTGTATGGCGGAGGTAATAT
TCGTATTTTATTAGCGAGAAGTTTTATTTGATTTCG
GTAGAGGTAGGGAGATTTTATGTTAGTTCGTCGG
GGGGAGCGGTAGCGAGAGTAGTTTTTTTCGCGGT
GAATGGGAAAGTGGGTGGGAGTTTACGAGAGGG
TTTTACGGTGTTTTGACGTGCGTTGTTATATGATAT
TTTCGTTGTTTCGAAATAGATATTTTAGTGTACGA
ATTATTAGAATTAAAATTTAGCGTATTTAAAATGA
TATATTTTTTATTAGAAGAGTTTCGTTTTAGGGTAT
TGTATGTTTTTGTAGATGTTTTTATTTTTAGTTTTAG
TAAATATACGTATAAGTT 3′</td></tr>
</table>

The primers were designed with Methyl Primer Express™ Software v1.0 (Applied Biosystems, USA)
